# Fate of Clinical Research Studies after Ethical Approval – Follow-Up of Study Protocols until Publication

**DOI:** 10.1371/journal.pone.0087184

**Published:** 2014-02-19

**Authors:** Anette Blümle, Joerg J. Meerpohl, Martin Schumacher, Erik von Elm

**Affiliations:** 1 Institute for Medical Biometry and Statistics, Center for Medical Biometry and Medical Informatics, University Medical Center, Freiburg, Germany; 2 Cochrane Switzerland, Institute of Social and Preventive Medicine (IUMSP), Lausanne University Hospital, Lausanne, Switzerland; Children's Hospital of Eastern Ontario, Canada

## Abstract

**Background:**

Many clinical studies are ultimately not fully published in peer-reviewed journals. Underreporting of clinical research is wasteful and can result in biased estimates of treatment effect or harm, leading to recommendations that are inappropriate or even dangerous.

**Methods:**

We assembled a cohort of clinical studies approved 2000–2002 by the Research Ethics Committee of the University of Freiburg, Germany. Published full articles were searched in electronic databases and investigators contacted. Data on study characteristics were extracted from protocols and corresponding publications. We characterized the cohort, quantified its publication outcome and compared protocols and publications for selected aspects.

**Results:**

Of 917 approved studies, 807 were started and 110 were not, either locally or as a whole. Of the started studies, 576 (71%) were completed according to protocol, 128 (16%) discontinued and 42 (5%) are still ongoing; for 61 (8%) there was no information about their course. We identified 782 full publications corresponding to 419 of the 807 initiated studies; the publication proportion was 52% (95% CI: 0.48–0.55). Study design was not significantly associated with subsequent publication. Multicentre status, international collaboration, large sample size and commercial or non-commercial funding were positively associated with subsequent publication. Commercial funding was mentioned in 203 (48%) protocols and in 205 (49%) of the publications. In most published studies (339; 81%) this information corresponded between protocol and publication. Most studies were published in English (367; 88%); some in German (25; 6%) or both languages (27; 6%). The local investigators were listed as (co-)authors in the publications corresponding to 259 (62%) studies.

**Conclusion:**

Half of the clinical research conducted at a large German university medical centre remains unpublished; future research is built on an incomplete database. Research resources are likely wasted as neither health care professionals nor patients nor policy makers can use the results when making decisions.

## Introduction

Patients and health professionals should be able to consider and appraise all the evidence available from medical research in order to make informed decisions about health issues. Such evidence on effectiveness and potential harm of health care interventions comes from interventional and observational studies, published in original articles and well-conducted systematic reviews summarizing primary studies. It has long been known that only a part of all clinical studies ultimately reaches the stage of full publication in peer-reviewed journals [1]. Publication or non-publication of studies is influenced by factors such as the nature and direction of their results [2]–[5]. The prevailing underreporting is wasteful and can result in biased estimates of treatment effect or harm [6]. Prospective trial registration has become an important measure to reduce underreporting by revealing studies that remained unpublished and hidden for the public. While Switzerland makes prospective registration of all human research studies mandatory from 2014 on (http://www.kofam.ch/en), this is still not the case in most jurisdictions including the European Union and the USA. Publication outcome is not only influenced by the direction of study results but also by characteristics such as study design and size, funding source or the presence of an international collaboration [7]. Main reasons for non-publication are lack of time or low priority, results not deemed important enough and journal rejection [8].

Consequently, only a particular share of the body of evidence is available to users of research data including other researchers, health professionals and patients. It is given undue prominence in the literature. This can lead to treatment recommendations that are at best inappropriate and at worst dangerous [9]. Selective publication has been deemed unethical, also from a normative point of view [10].

Submission to a research ethics committee (REC) or a funding agency is the earliest stage at which a planned study is documented in detail. We set out to assemble an unselected cohort of clinical studies that were approved by the REC of the University of Freiburg/Germany (Albert-Ludwigs-Universität). We aimed to characterize the clinical research being conducted, quantify its publication outcome and compare study protocols and corresponding publications for selected aspects.

## Materials and Methods

### Cohort of study protocols

We were granted access to the REC's files, which included the protocols of human research studies submitted for ethical approval, amendments, correspondence and other ancillary documents. A first analysis based on 299 protocols of studies of all designs approved in 2000 was published earlier [11]. For the present analysis, we completed the cohort of study protocols by adding those approved during the years 2001 to 2002. The definitive analysis is thus based on the study protocols approved during the three consecutive years 2000 to 2002. We chose this time period because it was both accessible in the REC's archives and long enough to allow for completion of the included studies. If a study protocol described two or more sub-studies, we regarded each as a separate study.

### Data collection and definitions

We used a standardised data extraction form (MS Access 2010™) to collect data on study characteristics from the study protocols, amendments (if any), the REC's application forms, and correspondence including study design, sample size, type of funding, single-/multicentre status, leading study centre and domestic/international study status. If conflicting information was found, we recorded the information of the most recent document in our database. If the information was not reported in any of the documents, we classified it as “unclear”. Data were extracted by one investigator. If the investigator in charge could not decide on how to extract data (e.g. when classifying study design), the issue was discussed with a second investigator to reach a consensus. All database entries were cross-checked by a second investigator.

We classified studies according to their design using an algorithm established earlier [11]. The categories were as follows: randomised controlled trials, non-randomised intervention studies, diagnostic studies, observational studies (incl. cohort, case-control, cross-sectional studies), uncontrolled studies, or laboratory studies (i.e. using human tissue or blood e.g. for genetic research). Funding sources were classified as commercial or non-commercial and information extracted separately. Commercial funding was defined as any direct financial support or provision of material (e.g. of the study drug) by a private for-profit company. We further extracted whether a private company was involved in the planning, management or data analysis of the study. We assumed such involvement if the study protocol was written by its staff or if one of the authors was affiliated with the company. Non-commercial funding was defined as financial or other support by governmental funding agencies, public or private foundations (unless clearly linked to a private company) or research funds of hospitals or academic institutions. We further classified studies as international or domestic. If at least one centre outside Germany participated in recruitment of participants, the study was considered international, otherwise domestic. We extracted the planned overall number of participants to be recruited (study size); if the protocol indicated a range of values we used the smallest value. Information on current study status was collected from correspondence with the applicants or other documents available to the REC.

### Identification of corresponding publications

We systematically searched the following electronic databases and platforms: Medline (platform Ovid, database Ovid MedlineR+Daily Update), Web of Science, Google Scholar, Current Contents Medizin including content by the publishers Hogrefe, Karger, Kluwer, Springer and Thieme (combined searches on the Medpilot platform www.medpilot.de) and the University's publication registry (Forschungsdatenbank Freiburg, http://forschdb.verwaltung.uni-freiburg.de/forschung). For randomised controlled trials, we also searched the Cochrane Central Register of Controlled Trials (issues 2/2010 - 4/2011), which contains records of controlled trials from Medline (quarterly updated), Embase (annually updated) and those identified by manual searches of journals that are not indexed in electronic literature databases [12]. A new search strategy was established for each study protocol including keywords from the protocol, such as experimental drug, study name or acronym, studied health condition or names of applicants. We used variants of search terms (e.g. synonyms) and additional search terms (e.g. trade names of drugs or devices) where appropriate. The search strategies were manually adapted to the specific syntax of each literature database. Searches for the protocols of the year 2000 were conducted between July 2011 and January 2012 and included an update of the earlier search conducted in 2006 [11]. For the protocols of the years 2001 and 2002, the searches were conducted between August 2009 and January 2010. We retrieved the full text of potentially eligible publications and set up an electronic library of pdf-documents linked to our MS Access 2010™ database. If we came across additional eligible references by other sources (e.g. reference lists of identified articles), we included them. Disagreements on eligibility were resolved by discussion and consensus among the authors. Only articles that contained at least some information on the study's objectives, methods and results and were published in a scientific journal were considered full publications. Review articles and published conference abstracts were excluded. Full reports of preliminary results published before completion of recruitment or data collection as planned were counted as full publications. Retrieved articles were read in full by one investigator. Key elements of study design and methods, but also study acronyms and names of authors, were used as criteria to decide whether the publication was considered matching a study protocol. Any uncertainties were discussed in regular group meetings.

In order to complement the electronic searches, we surveyed the investigators applying to the REC by writing personalised letters. In an appended questionnaire, we asked them for verification of the already identified publications and for references of additional publications we may have missed. We also asked whether the project (a) had been completed as planned (according to the protocol), (b) had been discontinued entirely or at the local study site, or (c) is still ongoing with or without continued recruitment or data collection. The letters and questionnaires were sent out in February 2010 and reminder letters in May 2010. Undeliverable letters were sent out again if the investigators' new address could be determined.

Based on the information from the survey, we checked and updated our publication database by deleting wrongly attributed references and adding any new. We also considered information on the current project status from other sources such as correspondence between the REC and investigators and information from publications. If the information from the survey did not match with what was reported in the publication and could not be clarified otherwise, we used the information from the publication. If we found a corresponding publication by our electronic searches, but received no response in the survey, we used the publication to determine the study's status.

### Data analyses

We used queries in MS Access 2010 and tabulation in Microsoft Excel 2010 to obtain standard descriptive statistics. We calculated the proportion of published study protocols (i.e. the proportion of studies that had been started at the local study site and resulted in at least one corresponding full publication), as well as its binomial 95% confidence interval. We used Pearson's χ^2^ test to examine associations between study characteristics and publication proportion and calculated McNemar odds ratios for disclosure of funding information in pairs of protocols and publications of commercially and non-commercially funded trials [13]. All comparisons were pre-planned. A p-value of 0.05 was used as threshold for statistical significance. For agreement of funding information between protocols and publications, we calculated Cohen's kappa values with 95% confidence intervals [14].

## Results

**Figure 1:**
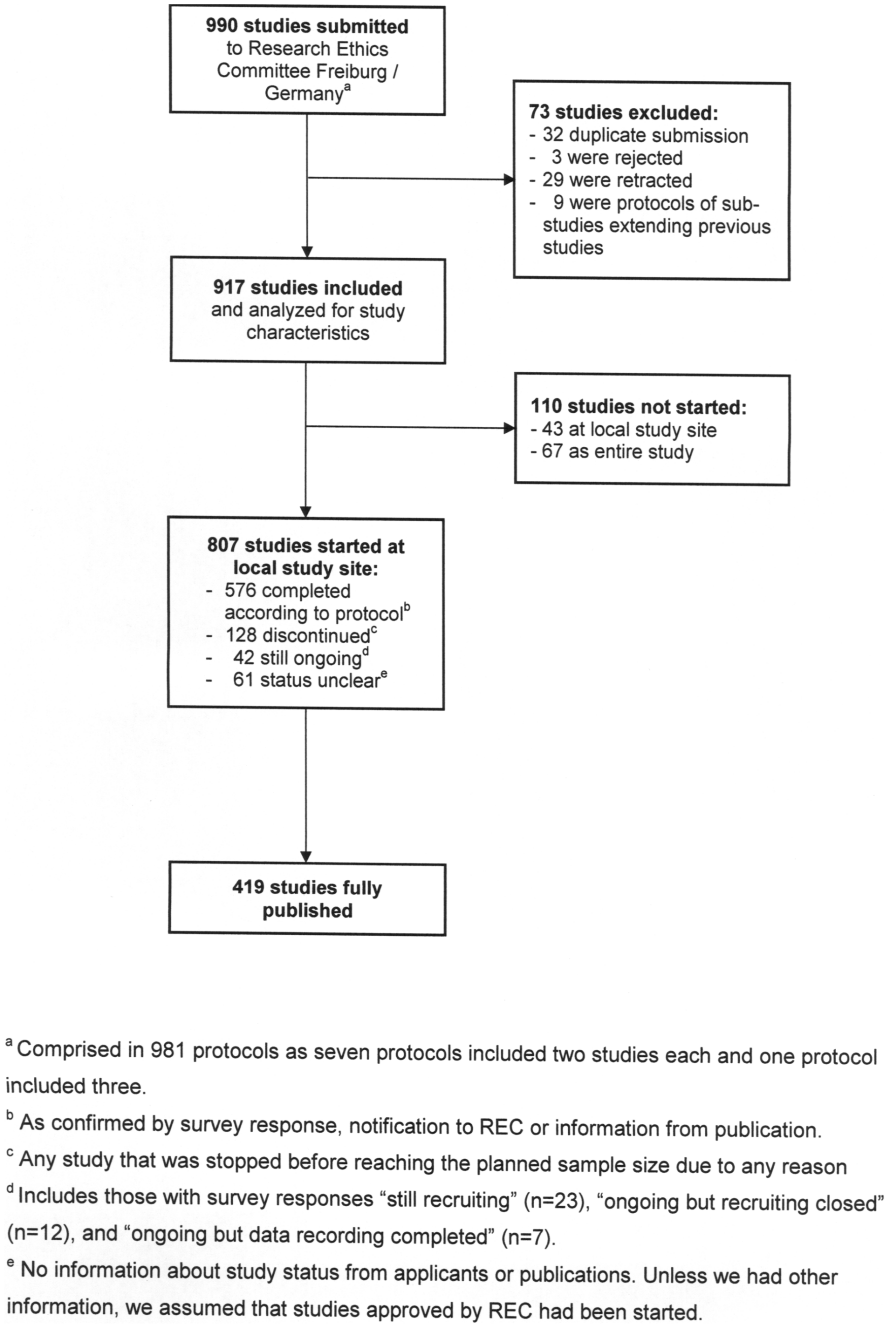
Flowchart of study protocols approved between 2000 and 2002 by the research ethics committee of the University of Freiburg/Germany with number and study status of included studies.

Between 2000 and 2002 the REC of the University of Freiburg approved 981 study protocols containing information on 990 individual studies (Figure 1). Seven protocols comprised two sub-studies and one comprised three sub-studies; we counted each sub-study separately. We excluded 73 studies because they were either duplicate submissions from several participating centres or the study was rejected, retracted or an extension of a previous study. Our final dataset comprised 917 approved studies.

### Characteristics of included studies

Almost half of the submitted studies were randomised controlled trials, which was the most frequent study design (408 studies, 45%). Of those, most were of parallel design (364 studies, 89%) with two treatment arms (269 studies) or three or more treatment arms (95 studies). Twenty-eight studies (7%) had a cross-over design and 16 (4%) another variant design, such as factorial or intra-individual comparison. The second most frequent study design were uncontrolled studies (186 studies, 20%), such as case series or uncontrolled phase I/II studies, followed by laboratory studies using human tissue or blood (138 studies, 15%), non-randomised intervention studies (72 studies, 8%), cross-sectional studies (42 studies, 5%), diagnostic studies (41 studies, 4%), comparative cohort studies (23 studies, 2%), case-control studies (6 studies, 1%), and one health services research study (0.1%) (Table 1).

**Table 1:** Publication status and characteristics of included studies.

Study characteristics	Approved (column %)	Started at local study site	Of those started:	
			Published (row %)	Not published (row %)
Total	917 (100)	807	419 (52)	388 (48)
**Study design**				
Randomised controlled trial	408 (45)	355	201 (57)	154 (43)
Non-randomised intervention study	72 (8)	65	33 (51)	32 (49)
Diagnostic study	41 (4)	36	21 (58)	15 (42)
Cohort study	23 (2)	19	8 (42)	11 (58)
Case-control study	6 (1)	6	3 (50)	3 (50)
Cross-sectional study	42 (5)	40	16 (40)	24 (60)
Uncontrolled study	186 (20)	163	75 (46)	88 (54)
Laboratory study	138 (15)	122	61 (50)	61 (50)
Health services research	1 (<1)	1	1 (100)	0
		*Pearson χ^2^ (df 8) = 10.173, p = 0.253*		
**Study size**				
Size≥median of 120	449 (49)	391	224 (57)	167 (43)
Size<median of 120	429 (47)	379	177 (47)	202 (53)
Unclear	39 (4)	37	18 (49)	19 (51)
		*Pearson χ^2^ (df 2) = 8.808, p = 0.012*		
**Collaboration**				
Single-centre study	383 (42)	340	159 (47)	181 (53)
Multi-centre study	534 (58)	467	260 (56)	207 (44)
		*Pearson χ^2^ (df 1) = 6.257, p = 0.012*		
Only multi-centre studies:				
International	310 (58)	276	173 (63)	103 (37)
Domestic	221 (41)	189	87 (46)	102 (54)
Unclear	3 (<1)	2	0	2 (100)
		*Pearson χ^2^ (df 2) = 15.124, p = 0.00052*		
Leading centre:				
Local	83 (15)	76	41 (54)	35 (46)
Other	448 (84)	388	218 (56)	170 (44)
Unclear*	3 (<1)	3	1 (33)	2 (67)
		*Pearson χ^2^ (df 2) = 0.74, p = 0.691*		
**Funding (as stated in protocol)**				
Commercial	422 (46)	368	203 (55)	165 (45)
Non-commercial	140 (15)	131	75 (57)	56 (43)
No funding stated	355 (39)	308	141 (46)	167 (54)
		*Pearson χ^2^ (df 2) = 7.695, p = 0.021*		
Only commercially funded studies:				
Sponsor involved	362 (86)	318	182 (57)	136 (43)
Sponsor not involved	60 (14)	50	21 (42)	29 (58)
		*Pearson χ^2^ (df 1) = 4.053, p = 0.044*		

*For two studies (one international, one domestic), the leading centre was not determined at the time of REC submission and for one international study there was no intention to define a leading centre.

The planned sample size was stated in 878 studies (96%) and ranged from 3 to 9300 participants (median, 120). The planned duration of enrolment was specified for 382 (42%) studies and ranged from less than 1 month to 120 months (median, 12 months). 383 studies (42%) planned recruitment in a single centre and 534 (58%) in multiple centres. Of the multi-centre studies, 310 (58%) included an international collaboration and 221 (41%) a collaboration with other centres in Germany. Eighty-three multi-centre studies (15%) were led by the local investigators and 448 (84%) by other study centres in Germany or abroad. For three studies, the collaboration status and leadership role remained unclear. Of the 221 domestic studies, 49 (22%) were led by the investigators in Freiburg and 171 (77%) by another study centre in Germany. For two studies (one international, one domestic), the leading centre was not determined at the time of REC submission and for one international study there was no intention to define a leading centre (all three grouped as unclear in Table 1).

Commercial funding was present in 422 studies (46%) according to protocol information (Table 1). In 60 of those, the sponsor provided study drugs or other material, but was not involved in study conduct otherwise. Information on non-commercial funding was given for 140 studies (15%), including applications for funding by the German Research Foundation (Deutsche Forschungsgemeinschaft) in 51 studies and the Federal Government (Ministry of Education and Research/Bundesministerium für Bildung und Forschung; Federal Ministry of Health/Bundesministerium für Gesundheit) in 26 studies.

### Course of studies and publication outcome

In the survey, we obtained responses for 825 of 917 approved studies (response rate 90%). Including information from other sources, the project status could be determined for 856 studies (93%): 807 (88%) were started at the local study site and 110 (12%) were not started, either locally or in all study centres (Figure 1). Of the 807 initiated studies, 576 (71%) were completed according to protocol, 128 (16%) discontinued and 42 (5%) still ongoing at the time of our study. The latter included studies that were still recruiting participants (n = 23), were ongoing after completed recruitment (n = 12) or were ongoing after completed data collection (n = 7). For 61 (8%) there was no information about current status and we assumed that they had been started, at least.

We identified 782 full publications that corresponded to 419 of the 807 studies. The year of publication ranged from 2000 to 2011. Consequently, the overall publication proportion was 52% (95% CI: 0.48–0.55). The median number of publications per study was 1 and the range was 1 to 56. Of the 807 initiated studies, 135 (17%) had more than one corresponding publication. Of note, one laboratory study was still ongoing eight years after ethical approval and had yielded a total of 56 publications until then. In the 770 initiated studies with information about the number of participants (not available for 37), it was planned to recruit at least 298,242 study participants overall (i.e. at all study sites). Of those, 178,254 (60%) participants had their data reported in publications corresponding to the 419 study protocols. In turn, 119,988 (40%) persons participated in the 388 studies that ultimately remained unpublished.

The publication proportion ranged from 40% (95% CI: 0.25–0.57) in cross-sectional studies to 58% in diagnostic studies (95% CI: 0.41–0.74) (Table 1). However, the differences by study design did not reach statistical significance (p = 0.253). In a post-hoc analysis we combined randomized and non-randomized interventional studies; the publication proportion was 56% (95% CI: 0.51–0.61). In contrast, in observational studies (combining cohort, cross-sectional and case-control studies) it was 42% (95% CI: 0.29–0.54). We further analysed whether study size, single or multi-centre status and type of funding were associated with full publication. Larger studies and multi-centre studies were more likely to be published than smaller studies and single-centre studies, respectively (both comparisons: p = 0.012) (Table 1).

In the group of multi-centre studies, the publication proportion of international studies (63%; 95% CI: 0.57–0.68) was higher than of domestic studies (46%; 95% CI: 0.39–0.53; p = 0.00052). Studies with any funding declared in the protocol (56%; 95% CI: 0.51–0.60) were more often published than studies without (46%; 95% CI: 0.40–0.52; p = 0.021). Thirty-two (63%) of the 51 studies funded by the German Research Foundation and 12 (46%) of 26 studies funded by the federal government were published.

Of the 419 studies with subsequent publications, evidence of commercial funding was present in the protocols of 203 (48%) and in the corresponding publications of 205 (49%) (Table 2). For most of these studies (339; 81%), information on presence or absence of commercial funding was in agreement between protocol and publications. Cohen's kappa was 0.62 (95% CI: 0.54–0.69). However, in 80 (19%) comparisons the funding status did not match: Commercial funding stated in the protocol was not reported in any of the corresponding publications for 39 studies. In turn, commercial funding reported in publications was not stated in the protocol for 41 studies (Table 2). Consequently, the ratio of counts of discordant pairs (McNemar odds ratio) was 1.05 (95% CI: 0.66–1.67).

**Table 2:** Funding status in protocols and corresponding publications - commercial funding.

		Information in publication, number of studies		
		Yes	No	Total
Information in protocol, number of studies	Yes	164	39	203
	No	41	175	216
	Total	205	214	419

Analogously, evidence of non-commercial funding was present in the protocols of 75 (18%) studies and in corresponding publications of 147 (35%) (Table 3). For most of the 419 studies (315; 75%), information on presence or absence of non-commercial funding was in agreement between protocol and publications. Cohen's kappa was 0.39 (95% CI: 0.28–0.49). In 104 (25%), the non-commercial funding status did not match: Non-commercial funding stated in the protocol was not reported in publications for 16 studies, and non-commercial funding reported in publications was not stated in the protocol for 88 studies (Table 3); the McNemar odds ratio was 5.50 [95% CI: 3.21–10.04]. In 40 publications (and none of the protocols) there was a statement of both commercial and non-commercial funding.

**Table 3:** Funding status in protocols and corresponding publications - non-commercial funding.

		Information in publication, number of studies		
		Yes	No	Total
Information in protocol, number of studies	Yes	59	16	75
	No	88	256	344
	Total	147	272	419

For protocols with two or more corresponding publications we regarded funding status as reported if it was found in at least one publication.

For publications with both commercial and non-commercial funding, both components were compared separately.

The predominant language of the publications was English: 367 (88%) studies were published in English and 25 (6%) in German. This predominance was found in both international and domestic studies, as well as multi and single centre studies (Table 4).

**Table 4:** Language of publication(s).

Collaboration	Number of published studies (column %)	Median of number of publications (Range)	Number of studies published in English (row %)	Number of studies published in German (row %)	Number of studies published in English and German (row %)	Number of studies with publications (co-) authored by local investigator*
Total	419 (100)	1 (1–56)	367 (88)	25 (6)	27 (6)	259 (62)
Single-centre study	159 (38)	1 (1–18)	129 (81)	16 (10)	14 (9)	158 (99)
Multi-centre study	260 (62)	1 (1–56)	238 (92)	9 (3)	13 (5)	101 (39)
*International*	*173 (67)*	*1 (1–56)*	*163 (94)*	*2 (1)*	*8 (5)*	*58 (34)*
*Domestic*	*87 (33)*	*1 (1–9)*	*75 (86)*	*7 (8)*	*5 (6)*	*43 (49)*
*Leading centre: Local*	*41 (16)*	*1 (1–56)*	*30 (73)*	*5 (12)*	*6 (15)*	*35 (85)*
*Leading centre: Other*	*218 (84)*	*1 (1–9)*	*207 (95)*	*4 (2)*	*7 (3)*	*65 (30)*
*Unclear*	*1*	*1*	*1 (100)*	*0*	*0*	*1 (100)*

* In a study with more than 1 publication local investigators were considered authors if they were authors of at least 1 publication.

We analysed whether local investigators (i.e. those submitting to the REC) were authors of subsequent publications. In 259 (62%) of the published 419 studies, local investigators were (co-)authors of at least one corresponding publication (Table 4). All but one publication from single-centre studies were authored by a local investigator. In this one publication, an expanded European data set was reported and the local investigator was acknowledged. Publications of 101 (39%) multi-centre studies were authored by a local investigator. In the subgroup of international multi-centre studies this proportion was 34% (Table 4). In multi-centre studies led by the local centre, the local investigators were authors in most studies (35; 85%), but less often (65; 30%) if the study was led by another centre.

## Discussion

We analysed clinical research projects approved by a German REC over three years, focusing on their publication outcome and the consistency of reporting in aspects such as funding. Only about half of the clinical studies that started recruiting participants were published as full articles about eight to ten years later. Study design was not associated with full publication. Multicentre status, presence of an international collaboration, large sample size, declared study funding and involvement of sponsor as stated in the protocol were positively associated with subsequent publication.

The Helsinki Declaration of the World Medical Association emphasizes that both authors and publishers of scientific research have ethical obligations and that negative and inconclusive results should be made publicly available, as is the case for positive results [15]. Our study confirms earlier evidence that the underreporting of clinical research is still prevalent [16]. It is sometimes put forward that more rigorous studies (e.g. randomised trials) will be published eventually while studies conducted with less methodological rigour may remain ‘in the file drawer’. In our cohort, study design was not associated with full publication; 43% of randomised trials had not been published.

It must be of concern that sizeable proportions of studies remain unpublished. Withholding research results pose several ethical problems since participants consent on the premise of contributing to the advancement of medical knowledge and considerable research resources are invested without any benefit in return. In our study, research results of almost 120,000 study participants remained hidden. Not only are patients who are willing to contribute to medical progress betrayed, but also public funds wasted. For instance, 19 of the 51 studies (37%) funded by the German Research Foundation and 14 of the 26 studies (54%) funded by the German federal government remained unpublished. Furthermore, non-publication and selective reporting of research results have an impact on the scientific knowledge. For instance, the conclusions of systematic reviews may be biased [17].

Information on sources of funding is important to appraise the validity of a study's results. It has been shown that commercially funded studies are more likely to produce favourable results and conclusions than those sponsored by other sources [18]. Although this information was consistent for most published studies, it is of concern that, firstly, for several studies with commercial funding or non-commercial funding, this information was omitted in the publications and, secondly, that funding sources are not always disclosed to the REC (provided that they are known at the time of submission). The discrepancy regarding funding information is consistent with our earlier finding in a sub-sample of randomised trials from the same cohort: There were important discrepancies in the eligibility criteria for trial participants between protocols and publications [19]. The present analysis found that commercial funding information was undisclosed in protocols and publications to the same extent. In contrast, the odds of finding information about non-commercial funding in the publication (but not the protocol) was 5.5 times higher than vice versa. A potential explanation is that industry involvement in the study's planning and conduct had already been determined at the time of writing of most commercially funded protocols, while in non-commercially funded trials, e.g. investigator-initiated trials, funding requests might be pending at this stage and consequently no funding information added to the protocol. Another reason may be that the publishing journals have strict policies for disclosure that incite investigators of non-commercially funded trials to disclose their funding sources more frequently.

Unsurprisingly, our results also show that most studies are published in English, even if the studies are domestic, multi- or single-centre studies with funding from a non-anglophone country, such as Germany. Given that language barriers continue to exist, in particular if new knowledge is to be transferred from research into practice, this must be of great concern. Likely, a sizable part of the healthcare communities not speaking English will not benefit from research findings reported in English language; concomitant efforts to provide translations (e.g. of summaries) are therefore needed [20], [21].

The problem of poorly reported or unreported study results has long been recognised, but is by far not resolved. Clinical trial registries can help to improve transparency and to inform patients, physicians and researchers about planned, ongoing and completed studies [22]. However, prospective registration is not mandatory for all types of clinical studies and the regulations differ between countries. In the United States “applicable clinical trials”, such as those on drugs, biological products and devices, have to be registered since 2007 (http://clinicaltrials.gov/ct2/manage-recs/fdaaa). In the European Union, clinical drug trials submitted to the European Medicine Agency (EMA) are registered in the EudraCT database, but only part of the information is open to the general public.

In Germany, trial registration is still optional and had not yet been introduced at the time of REC approval of the included trials. Therefore, we did not focus on this aspect in the present study. Analyses of more recent research will be able to address the impact of trial registration on publication outcomes more thoroughly.

The lack of access to key data of clinical trials has been put on the agenda of science policy makers and the public again by the recent “All Trials” initiative. This international initiative calls on governments, regulators and research bodies to implement measures to achieve that “all trials past and present should be registered, and the full methods and the results reported” (http://www.alltrials.net). Another recent effort called Restoring Invisible and Abandoned Trials (RIAT), calls on funders and investigators to publish or republish studies that were abandoned and left unpublished. The RIAT proposal provides authors with a set of criteria to assist with precise publication and republication of abandoned studies [23], [24]. Our empirical data underpins these efforts suggesting that the magnitude of underreporting has not diminished yet, despite joint large-scale initiatives such as trial registration.

Our comprehensive literature search employed several databases and was complemented by an investigator survey with a high response rate. We are confident that most full articles corresponding to the included study protocols could be identified. Despite these efforts, we cannot rule out that some were missed. Consequently, the publication proportion may be underestimated. On the other hand, we regarded several discontinued studies with published preliminary results as fully published, which could be perceived as an overestimation of the publication proportion. We excluded conference abstracts and other so-called ‘grey literature’ because those publications are often not indexed in electronic databases (in particular, abstracts of smaller conferences). Many of them are not found even by extensive literature searches and resulting estimates of publication outcome would therefore likely be incomplete or even biased. Further, we had to rely on several arbitrary definitions when extracting data and classifying studies. Since we included all types of studies submitted for ethical approval, we classified protocols by study design using a classification scheme that had proven useful in previous studies [7], [11]. Arguably, other criteria could have been used. For clinical trials, we decided against using the phase I to IV classification since it was not applied consistently in the included protocols. Alternative definitions would have been possible also for other study variables. However, given that all variables were defined *a priori* we are confident that our choices did not lead to any systematic error in our analyses. Clearly, it would be interesting to analyse more recent study protocols, as the quality of protocols and publications and the practices of scientific reporting change over time. In particular, trial registration has been introduced more widely since then. However, sufficient time must have elapsed before the ultimate fate of studies with regard to completion and publication can be determined. The obvious dilemma is that including more recent protocols would have left insufficient time for studies to be completed and results to be published [25]. In our sample, about five percent of studies were still ongoing eight to ten years after ethical approval. We chose to analyse the period from REC approval to publication because reliable data for both these time points were available. An estimate of the time elapsed between completion of the study (e.g. end of data collection) and publication would have been more meaningful. However, such information was not included regularly in study reports or REC files.

It would also have been interesting to investigate the reasons for non-publication. However, based on our prior experience with approaching local investigators for empirical research, we deemed that it is not feasible in a postal survey (in particular up to 10 years later) as it implies asking sensitive questions and likely would have influenced the response rate negatively. In fact, in many cases, non-publication of research has to do with poor project management, disagreements in research groups or other unforeseen events, and it is unlikely that trialists would have disclosed such circumstances in a survey.

We used a sample of studies conducted in various disciplines at a large German university. Many were multi-centric, international or both and studies could be included without seeking the trialists' consent. We are therefore confident that our results have some external validity in similar clinical research environments in other high-income countries.

## Conclusion

In a large unselected sample of clinical research projects approved by a German research ethics committee, only about half of the started studies were published. In addition, 16% of the started studies were discontinued. Crucial information such as study funding differed between protocols and publications in about 20% of published trials. If only part of the accumulated research data are accessible for those potentially interested, scarce research resources are wasted. Furthermore, health care professionals and patients cannot make decisions based on all the available evidence and other researchers may build future projects on an incomplete or even biased database.
